# Antipyretic and antinociceptive potential of extract/fractions of *Potentilla evestita* and its isolated compound, acacetin

**DOI:** 10.1186/1472-6882-14-448

**Published:** 2014-11-18

**Authors:** Abdur Rauf, Rehan Khan, Haroon Khan, Barkat Ullah, Samreen Pervez

**Affiliations:** Institute of Chemical Sciences, University of Peshawar, Peshawar, 25120 KPK Pakistan; Geology Department, University of Peshawar, Peshawar, KPK Pakistan; HEJ Research Institute, Institute of Chemical and Biological sciences, Karachi University, Karachi, Pakistan; Department of Pharmacy, Abdul Wali Khan University, Mardan, 23200 Pakistan; Department of Botany, University of Peshawar, Peshawar, KPK Pakistan; Department of Pharmacy, University of Peshawar, Peshawar, 25120 KPK Pakistan

**Keywords:** *Potentilla evestita*, Acacetin, Antipyretic, Peripheral and central antinociceptive activity

## Abstract

**Background:**

Fever and pain management is a very challenging job for the clinician as the available synthetic agents are causing serious side effects. The present research article deals with the antipyretic and antinociceptive activity of extract/fractions of *Potentilla evestita* and acacetin isolated from the chloroform fraction of the plant.

**Methods:**

Various chromatographic and spectroscopic techniques were used for the isolation and characterizion of compound. *In*-*vivo* yeast induced fibrile mice were used for antipyretic activity while acetic acid induced writhing and formalin tests were used for antinociceptive.

**Results:**

The extract/fractions of *P. evestita* caused marked antipyretic effect during various assessment times in which chloroform was the most prominent followed by ethyl acetate. When acacetin was injected, it produced marked effect with maximum activity of 33.28% and 55.01% at 5 and 10 mg/kg i.p respectively. When studied in acetic acid induced writhing test, the extract/fractions evoked significant antinociceptive effect in which chloroform was the most effective fraction followed by ethyl acetate. Acacetin showed significant antinociceptive effect with 44.77% and 67.03% reduction in abdominal constriction at 5 and 10 mg/kg i.p., respectively. Similarly, it evoked significant dose dependent reduction in noxious stimulation with 42.07% and 64.57% pain attenuation at 5 and 10 mg/kg i.p., respectively in initial phase. In the late phase, it illustrated more dominant effect with 46.32% and 67.29% reduction of painful sensation.

**Conclusions:**

In conclusion, the extract/fractions of *P. evestita* as well as the isolated compound, acacetin showed strong antipyretic and antinociceptive activity in various animal models possibly mediated through both peripheral and central mechanism.

## Background

*Potentilla evestita* belong to family Rosaceae. It is distributed in the eastern Himalayan range from Indus to Kumaon, in Arctic, Alpine and temperate regions of the Northern hemisphere. *P. evestita* is a small perennial flowering herb. It has long hairy leaves with divided rootstock [1, 2]. In Pakistan, it is usually found in Gilgit regions. In literature, several medicinal uses of *P. evestita* has been reported such as analgesic, antimicrobial, anti-inflammatory, anti-diarrheal, anti-diabetic, hepatoprotective, anticancer, antispasmodic and ulcerative colitis [1].

Phytochemically, approximately 43 compounds have been isolated from *P. evestita*. The rhizomes of *P. evestita* contains rich amount of tannins *i.e*. 3.5% hydrolysable tannins and 15-20% condensed tannins, pregnane derivative, 2,6 beta,7beta-trihydroxy-4-methyl-19-norpregna-1,3,5(10)-trien-17-one, and pregnane derivative, 11alpha,17alpha,21-trihydroxypregna-4,16(22)-diene-3,20-dione, have also isolated and reported from *P. evestita* [3, 4]. The *in*-*vivo* antinociceptive and anti-inflammatory activities of umbelliferone isolated from the chloroform fraction of *P. evestita* is already reported [5]. The antinociceptive/antiinflammtory activity of acacetin, a bioflavonoid is already present in literature. Most probably it act by interfering with 5-HT_1A_, GABA/BDZs and opioid receptors but not the NO-cGMP-K^+^ channel pathway [6]. However, the antipyretic activity of Acacetin has not yet been explored experimentally.

To the best of our knowledge based on the available literature, no such study carried out on the antipyretic and antinociceptive activity of the extract/fractions of *P. evestita*. Keeping in mind the potential ethnopharmacological uses of the plant in said conditions, the effect of extract/fractions of *P. evestita* was studied in yeast induced febrile test and antinociceptive activity. The bioactivity guided isolation led to the isolation of acacetin from the chloroform fraction of *P. evestita*. The acacetin was also subjected to yeast induced fever test and antinociceptive activity in both peripherally acting and centrally acting antinociceptive paradigms.

## Methods

### Plant specimen

The whole plant of *P. evestita* (15 kg) was collected from Gilgit, Pakistan. The plant was identified by Taxonomist Department of Botany, University of Karachi. Voucher specimen (voucher No. 707) has been deposited in the herbarium of the Department of Botany, University of Karachi, Karachi, Pakistan (Herbarium No. 71212).

### Extraction and Isolation

**Figure 1:**
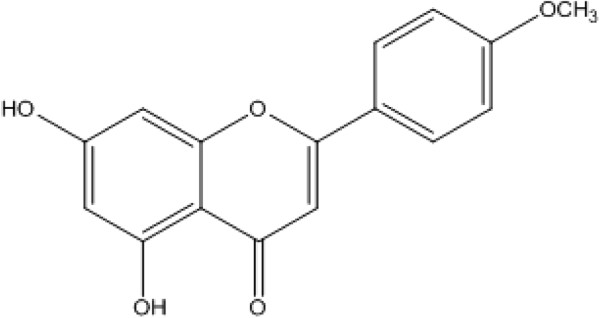
**Chemical structure of acacetin.**

Shade dried whole plant (15 kg) of *P. evestita* was ground into fine powder and soaked in 25 L ethanol for 10 days at room temperature. The resulting extract was filtered and the filtrate evaporated under reduced pressure at 45°C to yield 300 g dark brown residue. The residue was suspended in water and subsequently extracted with solvents of increasing polarity, namely *n*-hexane (3×10 L), chloroform (3×14 L), ethyl acetate (3×12 L) and methanol (1×3 L). Each extract, evaporated under reduced pressure to afford *n*-hexane fraction (70 g), chloroform (75 g) EtOAc extract (8 g), and methanol (40 g). The chloroform fraction (60 g) was subjected to Column chromatography on silica gel (Merck Silica gel 60 (0.063-0.200 mm), 5 × 60 cm). The column was first eluted with hexane-ethyl acetate (100:0 → 0:100) as the solvent system. A total of 100 fractions, RF-1 to RF-100 were obtained based on TLC profiles. Elution of the chromatogram with hexane- ethyl acetate (100:0 → 100:0) gave the isolation of a known compound, acacetin (80 mg). The structure (Figure 1) of compound was confirmed by comparing the NMR and physical data with the reported data in literature [7].

### Animals

BALB/c male mice (20–28 g) and Wistar rats (189–222 g) were used. Animals were kept under standard laboratory condition at 25 ± 2°C. They were fed laboratory diet *ad libitum* and allowed free access to drinking water under standard environmental conditions of temperature (25°C) in 12 h dark/12 h light control. The study was approved by the ethical committee of University of Karachi. Addtionally, all the experimental animals were treated according to ethical principles established by the University.

### Yeast induced fever test

The antipyretic activity was determined in albino mice. The animals were divided in groups (*n* = 6). All groups were fasted overnight and allowed free accesses drinking water. Group one received saline as control group, group second received paracetamol as standard drug while the remaining groups received extract/fractions of *P. evestita* (50 and 100 mg/i.p.) or acertain (5 and 10 mg/kg i.p.) The normal temperature was recorded using digital thermometer and then Fever was induced in all minces by injecting 20% aqueous suspension of Brewer’s yeast (10 ml/kg sc.). After 24 h rectal temperature was recorded and corresponding groups was injected with above doses. Rectal temperature was recorded periodically at 1, 2, 3, 4 and 5 h of drugs administration.

### Acetic acid induced writhing test

Albino mice (*n* = 6) weighing 20–28 g were used. All animals were withdrawn from food 2 h before the start of experiment and were divided in various groups. Group I was injected with normal saline (10 ml/kg) as control, Group II received standard drug diclofenac sodium (5 and 10 mg/kg) while the remaining groups were injected extract/fractions of *P. evestita* (50 and 100 mg/i.p.) or acertain (5 and 10 mg/kg i.p.). After 30 min of the above treatment animals were treated i.p. with 1% acetic acid. The number of abdominal constrictions (writhes) was counted after 5 min of acetic acid injection for the period of 10 min [8, 9].

### Formalin test

In the formalin test, male wistar rats (189–222 g) were used following our previously reported method [10]. Briefly, the prescreened animals were arranged into groups (*n* = 6) which received either saline (10 ml/kg), compound (5 and 10 mg/kg i.p.). For the induction of pain, 0.05 ml of formalin (2.5% formaldehyde) was injected into the plantar surface of the right hind paw, 30 min after the treatment of all the animal groups, as described above. The nociceptive response was considered as the time spent by rat walking or can stand on injected paw; partially elevated paw; total elevation of injected paw, injected paw licking or biting. The first 0–5 min was computed as the first phase (neurogenic) and 25–30 min as last phase (anti-inflammatory) in the assay. Tramadol^R^ (30 mg/kg i.p.) was used as a standard drug.

### Statistical analysis

Values are shown as mean values ± SEM of at least six animals. One-way analysis of variance was used for comparison test of significant differences among groups followed by Dunnet’s multiple-comparison post test. *P* < 0.05 was considered as significant from control.

## Results

### Effect of extract/fractions and acacetin in yeast induced fever test

**Figure 2:**
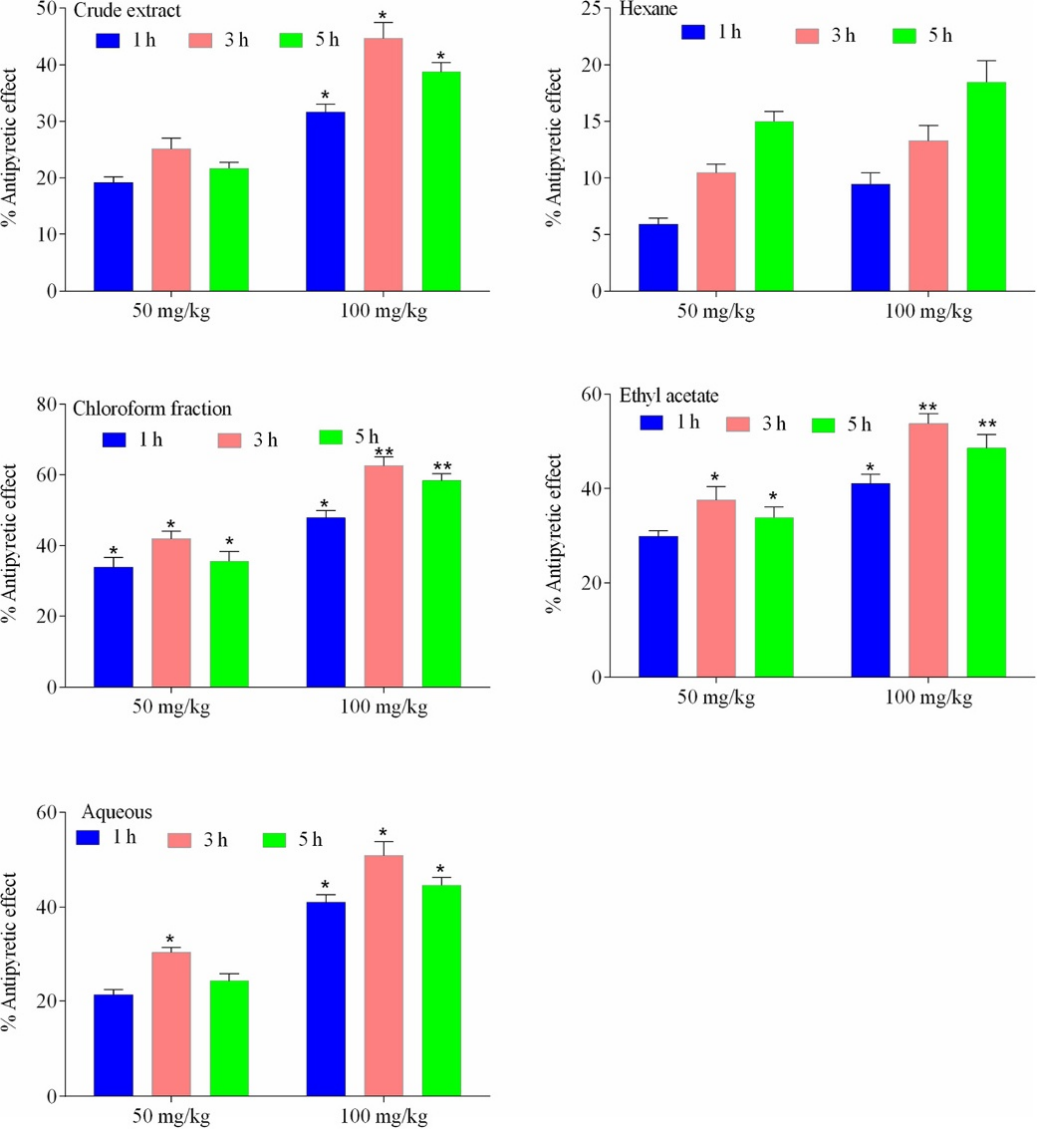
**Antipyretic activity (%) of crude extract/fractions in yeast induced fever test in mice.** The data were analyzed by analysis of variance followed by Dunnett’s test. Asterisks indicated statistically significant values from the control. *p < 0.05, **p < 0.01.

**Figure 3:**
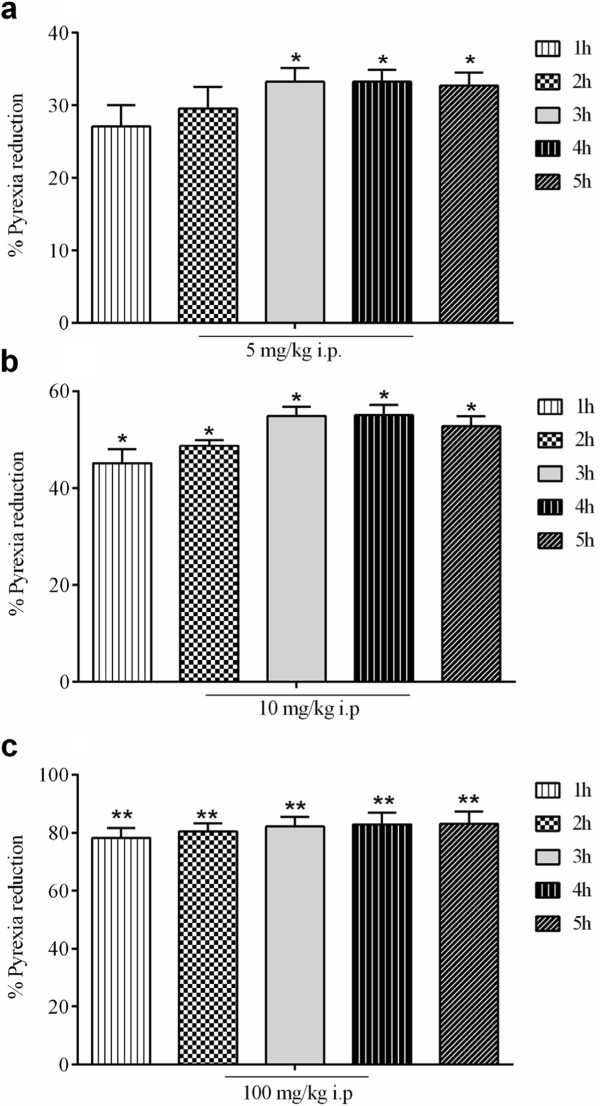
**Antipyretic activity (%) in yeast induced fever test in mice.** **[a]** Pretreatment of acacetin with 5 mg/kg i.p. **[b]** with 10 mg/kg i.p. **[c]** and Paracetamol 100 mg/kg i.p. The data were analyzed by analysis of variance followed by Dunnett’s test. Asterisks indicated statistically significant values from the control. *p < 0.05, **p < 0.01.

The percent antipyretic effect of extract/fractions of *P. evestita* is shown in Figure 2. The crude extract demonstrated significant attenuation of induced fever in dose dependent manner during various assessment times with maximum effect 3^rd^ of treatment. Upon fractionation, marked changes in effect were noted. Of the test articles, chloroform fraction was the most dominant fraction followed by ethyl acetate fraction; illustrated maximum effect after 3^rd^ h of treatment. The percent antipyretic effect of compound at test doses in yeast induced fever test is presented in Figure 3. Post-treatment of compound showed significant effect after 3^rd^ h of treatment that remained significant up to 5^th^ h (Figure 3a). At 10 mg/kg i.p., the antipyretic effect was significant even after 1^st^ h of drug administration that remained significant up to 5^th^ h (Figure 3b). However, the standard drug, paracetamol showed most prominent effect during various assessment times (Figure 3c).

### Effect of extract/fractions and acacetin in acetic acid induced writhing test

**Figure 4:**
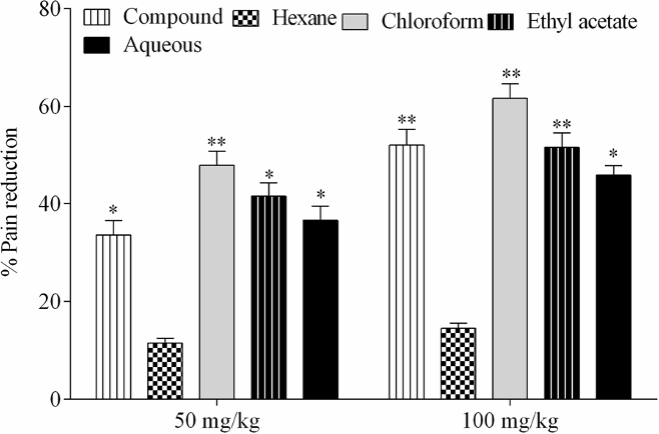
**The effect (%) of crude extract/fractions in acetic acid induced writhing test (** ***n*** **= 6).** The data were analyzed by analysis of variance followed by Dunnett’s test. Asterisks indicated statistically significant values from the control. *p < 0.05, **p < 0.01.

**Figure 5:**
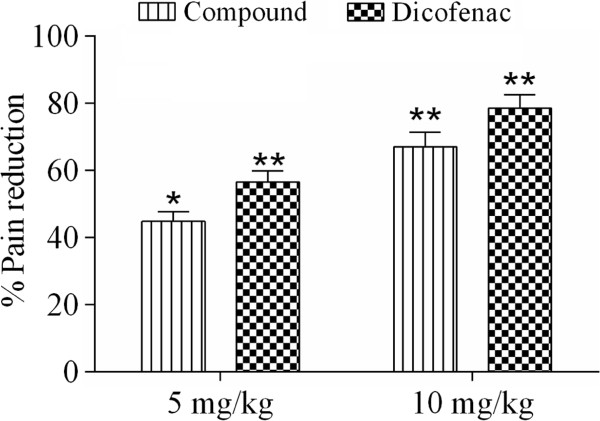
**The effect (%) of acacetin in acetic acid induced writhing test (** ***n*** **= 6).** The data were analyzed by analysis of variance followed by Dunnett’s test. Asterisks indicated statistically significant values from the control. *p < 0.05, **p < 0.01.

The antinociceptive effect of extract/fractions of *P. evestita* in acetic acid writhing test is presented in Figure 4. The crude extract provoked significant effect in dose dependent manner. However, fractionation produced marked changes in effect in which chloroform was the most prominent fraction followed by ethyl acetate. When acacetin was studied, it reduced pain perception in a concentration dependent manner. The reduction in abdominal constriction was 44.77% and 67.03% at 5 and 10 mg/kg i.p., respectively. However, the standard compound, diclofenac exhibited more dominant effect with 55.56% and 78.45% at 5 and 10 mg/kg i.p., respectively (Figure 5).

### Effect of acacetin in formalin test

**Figure 6:**
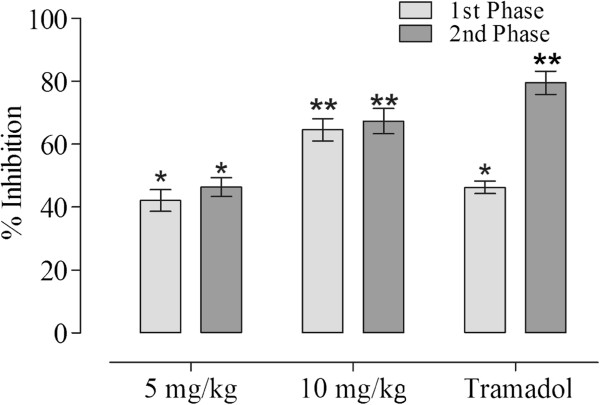
**Protection (%) of the acacetin in formalin induced flinching behaviour (** ***n*** **= 6).** The data were analyzed by analysis of variance followed by Dunnett’s test. Asterisks indicated statistically significant values from the control. *p < 0.05, **p < 0.01.

The formalin-induced flinching behaviour was significantly attenuated by the injection of compound in the first phase (Figure 6). It produced significant dose dependent reduction in noxious stimulation with 42.07% and 64.57% pain reduction at 5 and 10 mg/kg i.p., respectively. In the late phase, it demonstrated more dominant effect with 46.32% and 67.29% blockade of painful sensation.

## Discussion

The present study deals with the effect of crude extract/fractions of *P. evestita* and bioactive activity guided isolation led to the isolation of acacetin from the chloroform soluble fraction followed by test in antipyretic and antihyperalgesic effects.

It is well established that the subcutaneous injection of brewer’s yeast induces fever by increasing the synthesis of prostaglandin. It is considered as a useful test for the screening of plants materials as well as synthetic drugs for their antipyretic effect. The yeast-induced fever is called pathogenic fever and its etiology could be the production of prostaglandins [11, 12]. The inhibition of prostaglandin synthesis could be the possible mechanism of antipyretic action as that of paracetamol and the inhibition of prostaglandin can be achieved by blocking the cyclooxygenase enzyme activity [5]. There are several mediators for fever and the inhibition of these mediators is responsible for the antipyretic effect [11, 13, 14]. The extract/fractions of the plant caused significant amelioration of the fribile mice in a dose dependent manner. Among the fractions tested, chloroform was the most effective one followed by ethyl acetate. The intraperitoneal administration of isolated compound, strongly attenuated rectal temperature of yeast induced febrile mice. Thus it strongly complimented the antipyretic effect of extract/fractions of the plant and could be assumed that it interfered with the release or inhibit their activity of prostaglandins.

Of several *in*-*vivo* tests available for the evaluation of antinociceptive activity of natural compounds, two commonly used (acetic acid induced writhing and formalin induced flinching behavior) were employed [15]. The algesic mechanism for acetic acid induced writhing test has been characterized as liberation of different endogenous mediators [13, 16, 17]. The injection of acetic acid produced nociception in the form contraction of the abdominal muscle accompanied by an extension of the forelimbs and body elongation. The extract/fractions of *P. evestita* exhibited profound antinociceptive effect in a dose dependent manner; with chloroform fraction as the most prominent fraction followed by ethyl acetate. The isolated compound significantly attenuated the abdominal constriction provoked by the acetic acid in a dose dependent manner. Thus one possible mechanism of antinociceptive activity of the extract/fractions of the plant as well as the isolated compound could be due to the blockade of the effect or the release of endogenous substances (arachidonic acid metabolites) that excite pain nerve endings.

In order to elucidate possible mechanism, the acetic acid induced writhing test is deficient in specificity; as different mechanism may be implicated in the reduction of muscular constriction for instance, sympathetic system through the release of biogenic amines, cyclooxygenases and their metabolites inhibition and through opioids receptors mechanisms [18]. This dearth was resolved while using the formalin test. This chemically induced noxious protocol is frequently used as a primary behavioral screening test for the assessment of the antinociceptive activity of compounds used in moderate, long lasting clinical pain [19]. Post formalin injection test produced a distinct biphasic response. The initial neurogenic phase (0–5 min) arises within seconds of formalin injection as a direct result of chemical stimulation of peripherally localized TRPA-1 containing nociceptors [10, 15]. The later tonic phase (25–30 min) occurs as a result of increased primary afferent drive with subsequent sensitization of nociceptive spinal neuron. It is observed that different analgesics may act differently [11, 20]. The centrally acting drugs such as opioid inhibit both phases equally but peripherally acting drugs, such as cyclooxygenase inhibitors inhibit only the late phase [21, 22].

The results of compound demonstrated strong antinociceptive in post-formalin induced flinching behaviour test. The effect was significant in both 1^st^ and 2^nd^ phases. Thus it is concluded that the peripheral effects were augmented by the central effects of the compound. However, details studies are most warranted to ascertain it further clinical utilization.

## Conclusions

Finally, the extract/fractions of *P. evestita* showed marked antipyretic and antinociceptive effect in animal models which strongly augmented by the isolated compound, acacetin from the chloroform fraction. Thus our study provided scientific rationale to the folk uses of the plant as antipyretic and analgesic. Additionally, acacetin is a new candidate for further detail studies to ascertain its clinical use in further detail studies.
